# Lens Extrusion from *Laminin Alpha 1* Mutant Zebrafish

**DOI:** 10.1155/2014/524929

**Published:** 2014-01-15

**Authors:** Mallika Pathania, Elena V. Semina, Melinda K. Duncan

**Affiliations:** ^1^Department of Biological Sciences, University of Delaware, 327 Wolf Hall Newark, DE 19716, USA; ^2^Department of Cell Biology, Neurobiology and Anatomy, Medical College of Wisconsin, Milwaukee, WI 53226, USA

## Abstract

We report analysis of the ocular lens phenotype of the recessive, larval lethal zebrafish mutant, *lama1* ^*a69/a69*^. Previous work revealed that this mutant has a shortened body axis and eye defects including a defective hyaloid vasculature, focal corneal dysplasia, and loss of the crystalline lens. While these studies highlight the importance of laminin *α*1 in lens development, a detailed analysis of the lens defects seen in these mutants was not reported. In the present study, we analyze the lenticular anomalies seen in the *lama1* ^*a69/a69*^ mutants and show that the lens defects result from the anterior extrusion of lens material from the eye secondary to structural defects in the lens capsule and developing corneal epithelium associated with basement membrane loss. Our analysis provides further insights into the role of the lens capsule and corneal basement membrane in the structural integrity of the developing eye.

## 1. Introduction

The ocular lens is a transparent, avascular tissue made of two polarized cell types, the lens epithelial cells, and the lens fiber cells, which are completely surrounded by a specialized basement membrane, the lens capsule. The capsule is secreted by the cells it surrounds and is composed of laminin, collagen IV, entactin/nidogen, and heparan sulfate proteoglycans including perlecan [1–3]. Like all basement membranes, the lens capsule serves as an extracellular depot for growth factors and proteases [4] while also directly binding to cellular receptors such as integrins [5, 6] to provide signals which control the phenotype of the attached cells [7]. The capsule also serves as a selectively permeable barrier between the lens and the ocular environment [8], protecting the lens from infection while also conferring immune privilege [9]. Finally, the lens capsule is important for lens structural integrity and serves as the attachment site between the lens and the zonules, which suspend the lens in the correct location within the eye [10, 11] and transmit the forces necessary for accommodation in primates [12]. Consistent with these functions, mutations in genes encoding either lens capsule components [13, 14] or proteins necessary for lens capsule assembly [15–17] lead to diverse lens dysplasias [18, 19].

Laminin is an extracellular matrix (ECM) component secreted as a heterotrimer of *α*, *β*, and *γ* subunits. Currently, 16 different laminin heterotrimers have been identified; each comprised of a different combination of the five known *α*, four known *β*, and three known *γ* subunits [20, 21]. The lens capsule has been reported to contain laminin *α*1, *α*5, *β*1, *β*2, and *γ*1 [22], and mutations in human LAMB2 results in Pierson's syndrome, which is characterized by severe kidney disease associated with multiple ocular abnormalities including lens malformations and cataracts [23]. Notably, deletion of the* lama1*, *lamb1*, and *lamc1* genes result in postimplantation lethality in mice, apparently because laminin 111, the heterotrimer composed of laminin *α*1, *β*1, and *γ*1, is critical for the initial assembly of epithelial basement membranes [24]. Further, mutations have been identified in the zebrafish *lama1* (bashful; bal), *lamb1 *(grumpy; gup), and *lamc1* (sleepy; sly) genes, all of which result in profound body axis and brain defects [25–27].

Zebrafish mutations in the *lamb1* and *lamc1* genes also result in retinal lamination defects, as well as severe lens defects by three days after fertilization including the ectopic position of the lens within the retina, loss of lens capsule integrity, and inappropriate localization of the zebrafish lens marker ZL-1. By five days after fertilization, the lens has fragmented and is largely lost from the eye [18]. Mutations and morpholino driven knockdown of the *lama1* gene result in similar lens degeneration/loss although the phenotype appears more severe with the first defects apparent by 30 hpf while the lens is absent by 72 hpf leading to the conclusion that fiber cell morphogenesis was disrupted. While these studies make it apparent that the laminin 111 heterotrimer is critical for eye and lens development and function, none of the prior studies on these laminin mutants characterized these lens defects further. Here we reevaluate the lens phenotype of the zebrafish *lama1* mutant, *lama1* ^*a69*^, and find that the loss of the lens occurs upon its extrusion through the developing cornea suggesting roles for laminin 111 in the structural integrity of the eye.

## 2. Materials and Methods

### 2.1. Zebrafish Husbandry and Identification of the *Lama1* ^*a69*^ Mutant

The *lama1* ^*a69*^ zebrafish mutant was previously isolated in a forward genetic screen for ocular phenotypes and originally named *a69* [28] and then renamed *bal* ^*a69*^ when *a69* was found to be allelic to the *bashful (bal)* mutation by complementation [27]. The causative mutation for the phenotype was identified in the *lama1* gene [26] and the allele is now denoted *lama1* ^*a69*^ according to the 2013 Zebrafish Nomenclature Guidelines https://wiki.zfin.org/display/general/ZFIN+Zebrafish+Nomenclature+Guidelines. All mutant embryos die by 12 days after fertilization [26]. Control embryos were obtained as a product of the *lama1* ^*a69*^ mating scheme. All zebrafish (*Danio rerio*) were raised and maintained on a 14-h light/10-h dark cycle at 28.5°C. Embryos were obtained by natural spawning and their developmental stage was determined by time and morphological criteria. All experiments were conducted in accordance with the guidelines set forth by the Animal Care and Use Committees at the Medical College of Wisconsin and the University of Delaware.

### 2.2. Immunofluorescence

All fluorescent immunolocalization studies were performed as previously described, with a minimum *n* = 6 [29]. Briefly, both mutant and wild type embryos were collected and embedded in fresh Optimum Cutting temperature media (OCT, Tissue Tek, Torrance California). Sixteen micron thick sections were prepared on a cryostat and mounted on ColorFrost plus slides (Fischer Scientific, Hampton, New Hampshire). Sections were fixed by immersion in ice cold 1 : 1 acetone-methanol for 10 minutes at −20° Celsius and blocked with 2% BSA in 1X PBS for one hour at room temperature. This was followed by incubation with appropriate dilution of primary antibody (see below) in blocking buffer for 1 hour at room temperature. Two, 10-minute washes with 1X PBS were performed and unlabeled primary antibodies were detected with the appropriate AlexaFluor 568 or AlexaFluor 488 labeled secondary antibody (Life Technologies, Carlsbad, California) diluted 1 : 200 in blocking buffer containing a 1 : 2000 dilution of the nucleic acid stain Draq-5 (Biostatus Limited, Leicestershire, United Kingdom). Slides were visualized with a Zeiss LSM 780 confocal microscope configured with an Argon/Krypton laser (488 nm and 561 nm excitation lines) and Helium Neon laser (633 nm excitation line) (Carl Zeiss Inc., Göttingen, Germany). All comparisons of staining intensity between specimens were done on sections stained simultaneously and the imaging for each antibody was performed using identical laser power and software settings to ensure validity of intensity comparisons. In some cases, brightness and contrast of the images presented here was adjusted for optimum viewing on a computer screen, but in each case, care was taken to make similar adjustments in the mutant and control images.

Rabbit polyclonal antibodies against laminin were obtained from Abcam (Cambridge, Massachusetts) (cat no. 11575-250, raised against laminin from EHS tumor) and Sigma-Aldrich (Saint Louis, Missouri) (cat no. L9393, raised against laminin from EHS tumor) and used at a dilution of 1 : 200. A mouse monoclonal antibody recognizing the zebrafish lens (ZL1) was obtained from Zebrafish International Resource Centre (Eugene, Oregon) and used at a dilution of 1 : 500. A rabbit polyclonal antibody against Aquaporin 0 was obtained from EMD Millipore (Billerica, Massachusetts) (cat no. AB3071) and used at a dilution of 1 : 200. An anti-TGFbi (BIGH3) rabbit polyclonal antibody (cat no. 28660) was obtained from Santa Cruz Biotechnology (Santa Cruz, California) and used at 1 : 50 dilution. A rabbit polyclonal antibody to Collagen IV was obtained from Abcam (Cambridge, Massachusetts) (cat no. ab 6586) and used at a dilution of 1 : 200.

## 3. Results and Discussion

Basement membranes (BM) play diverse roles in vertebrates which include serving as a selectively permeable barrier between cells and the extracellular environment [30], providing signals that allow cells to sense their extracellular environment and respond by changing/maintaining cellular phenotype/behavior [31], the maintenance of an extracellular depot of growth factors/matricryptins [32], and the preservation of tissue structural integrity [33, 34]. The lens capsule, an unusually thick BM (7–48 *μ*m depending on age, genetic background, region measured, and species [35]) has been proposed to have all of these functions [1, 36], although the contribution of different BM components to these diverse roles has not been comprehensively investigated.

Laminins are heterotrimeric molecules that are found in all BMs that appear to provide the primary scaffolding necessary to assemble other BM components such as collagen IV, nidogen/entactin, and heparan sulfate proteoglycans into a fully functional ECM [37–41]. The human lens capsule has been reported to contain laminin *α*1, *α*5, *β*1, *β*2, and *γ*1 chains [22, 42] while these were also found to be the most abundant laminin mRNAs expressed by the embryonic mouse lens by RNAseq [43], thus the lens capsule has the potential to contain laminin 111, laminin 121, laminin 511, and laminin 521 heterotrimers [44]. No human diseases have been associated with mutations in *LAMA1*, *LAMA5*, and *LAMC1* (encodes laminin *γ*1) to date, although *lama1*, *lama5*, *lamb1*, and *lamc1* null mice are embryonic lethal [24, 45, 46], while a hypomorphic allele of *lama1* results in retinal defects in mice [47], point mutations in *LAMB1* result in lissencephaly-5 in humans [48], and mutations of *LAMB2* result in Pierson syndrome [49], which causes severe nephrosis and ocular abnormalities including lens malformations and cataracts demonstrating the critical role that these laminins play in development.

In zebrafish, mutations in the *lama1*, *lamb1*, and *lamc1* genes all result in a variety of severe defects in the notochord, body axis, muscle formation, and nervous system development. Notably, mutation or knockdown of any of these genes also results in a variety of ocular phenotypes including defects in retinal lamination, corneal defects, and lens malformations/degeneration although the timing and severity of the phenotype vary between alleles [21, 26, 50]. Previous studies of *lama1* ^*a69/a69*^ mutant embryos have shown that the lenses are profoundly abnormal with severe lens degeneration leading to the speculation that the lens epithelium and fiber cells did not differentiate normally [26]. In order to further clarify the role of laminin in lens development, here we carry out a more detailed analysis of the morphological and molecular consequences of the *lama1* ^*a69/a69*^ mutation on the lens.

### 3.1. *Laminin Alpha 1* Mutation Leads to Loss of Laminin Immunoreactivity in the Lens Capsule

**Figure 1:**
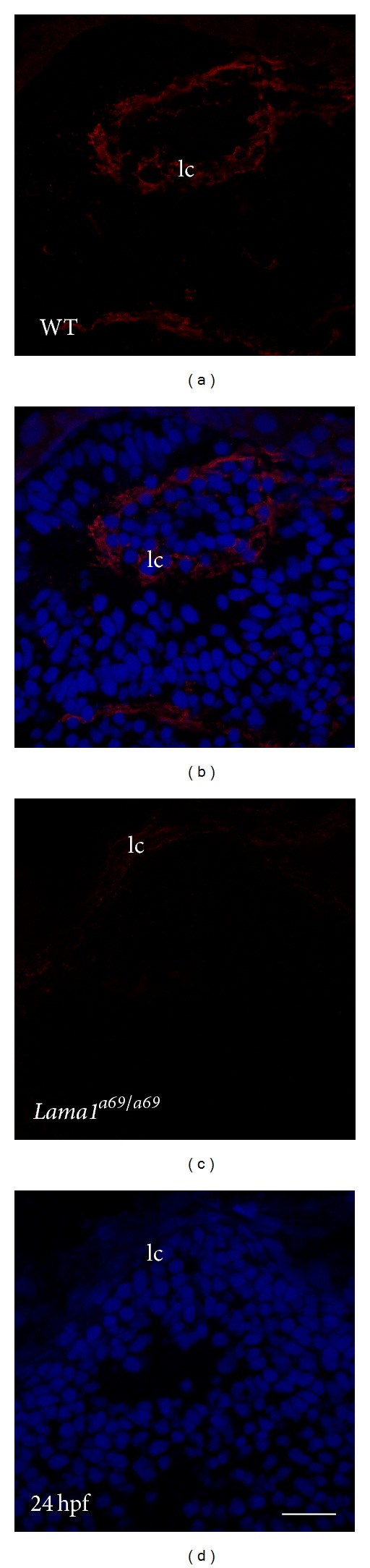
Laminin levels are downregulated in *lama1* ^*a69/a69*^ mutants. Immunofluorescent confocal microscopy showing laminin protein expression at 24-hpf. Eye from a wild type zebra fish embryo (a, b) showing normal distribution of laminin in the lens capsule at this stage. Eye from a *lama1* ^*a69/a69*^ mutant embryo showing downregulation of laminin expression (b, d). Laminin: red: DNA/Draq5: blue. lc: lens capsule. Scale bar = 35 *μ*m.

The zebrafish lens forms when a region of the head ectoderm thickens at 18 hours after fertilization (hpf) to form a ball of cells that delaminates from the overlying cell sheet between 20 and 24 hpf, at which time the lens epithelium and fiber cells are already apparent [51]. Laminin is found at all stages of this process as it is a component of the BM underlying the head ectoderm at 16 hpf and completely surrounds the newly delaminated lens at 24 hpf (Figures 1(a) and 1(b)) [51]. In contrast, the *lama1* ^*a69/a69*^ zebrafish lens exhibits little to no immunoreactivity against two different pan-laminin antibodies at 24 hpf (Figures 1(c) and 1(d), data not shown). This loss of laminin from the lens capsule likely occurs because the C56S mutation responsible for the *lama1* ^*a69*^ mutant phenotype is expected to disrupt one of the disulfide bridges necessary for laminin heterotrimer assembly [21, 52, 53], while assembly of the laminin heterotrimer is required for its secretion and assembly into the BM [54]. This suggests that the laminin 111 or laminin 121 networks are the main laminin heterotrimers present in the zebrafish lens capsule at this age. This is consistent with the prior detection of laminin 111 in the embryonic zebrafish lens capsule [18] and the known preference for laminin 111 in embryonic epithelial basement membranes [55, 56]. However, since both of the antibodies used here are raised against EHS-laminin, which is composed of laminin 111, it is still possible that other laminin heterotrimers such as laminin 511 and 521, which are likely components of mammalian lens capsules, are present, but not detected.

### 3.2. *Lama1* ^*a69*/*a69*^ Mutant Zebrafish Lenses Have Defects in Collagen IV Organization and Secretion

**Figure 2:**
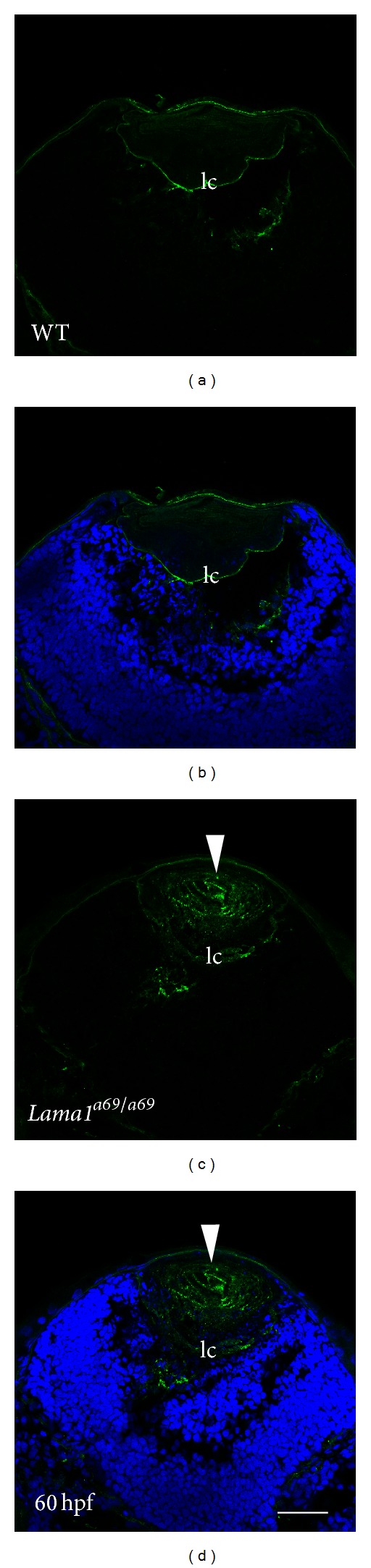
Collagen IV aggregates are seen in *lama1* ^*a69/a69*^ mutant lens fibers. Immunofluorescent confocal microscopy showing collagen IV protein expression at 60 hpf. Eye from a wild type zebrafish embryo (a, b) showing normal distribution of collagen IV in the lens capsule at this stage. Eye from a *lama1* ^*a69/a69*^ mutant embryo showing downregulation of collagen IV expression in the lens capsule, while Collagen IV retention is seen in the lens fibers (c, d arrowheads). Collagen IV: green; DNA/Draq5: blue. lc: lens capsule. Scale bar = 35 *μ*m.

Collagen IV is another heterotrimeric molecule ubiquitous to BMs including the lens capsule [57], integrating with the laminin scaffold to provide stability and strength to the basement membrane [1, 54]. Since the lens capsule was found to be nearly absent from *lamc1* mutant zebrafish [18], we investigated whether collagen IV was correctly assembled around* lama1* ^*a69/a69*^ mutant lenses. At 60 hpf, the wildtype lens was completely surrounded by a well formed collagen IV matrix while little to no staining was detected outside of the capsule (Figures 2(a) and 2(b)). In contrast, collagen IV was not found in this sharply demarcated distribution in *lama1* ^*a69/a69*^ mutants, instead, most of the staining was found within the lens, in a distribution consistent with the presence of collagen IV aggregates (Figures 2(c) and 2(d)). Notably, mice mutant for *lamc1*, which do not form the initial laminin 111 network which is normally found in the epiblast, also do not form an organized collagen IV network; instead, collagen IV was detected in aggregates throughout the embryo [46]. This suggests that the lens, like the early embryo, requires a laminin 111 scaffold for the appropriate assembly of the lens capsule. This loss of collagen IV organization is likely to contribute to the phenotype of these lenses as mutations in the *COL4A1* gene cause anterior segment defects [58, 59], while mutations in the *COL4A3* or *COL4A4 *genes result in Alport Syndrome in humans, which is associated with anterior and posterior lenticonus, capsular ruptures, and cataracts [59–64].

### 3.3. *Laminin Alpha 1* Mutation Does Not Disturb Fiber Cell Marker Expression but Leads to Lens Extrusion from the Eye

**Figure 3:**
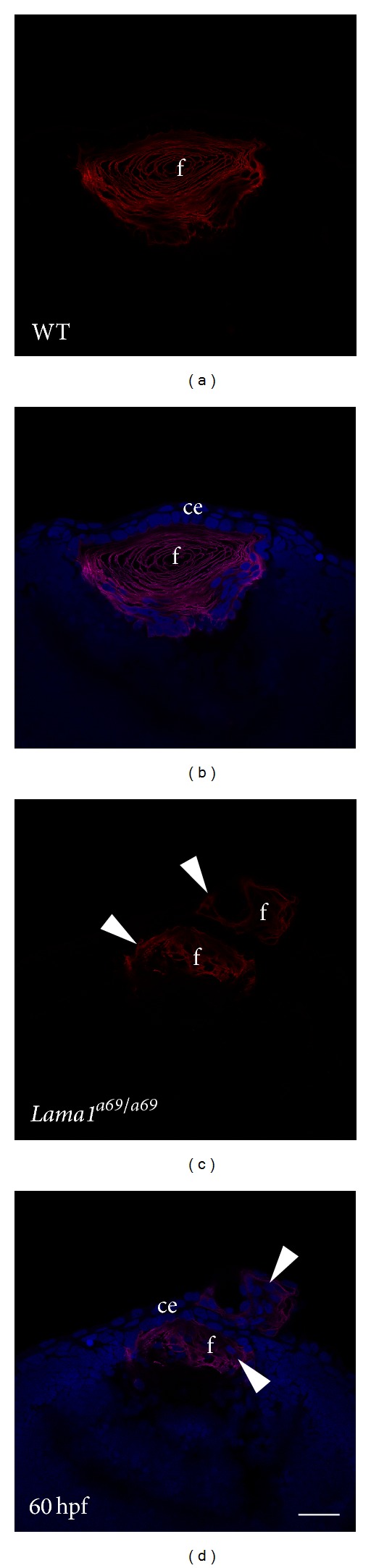
Lens fibers extrude from the eye in *lama1* ^*a69/a69*^ mutant zebrafish. (a, b) immunofluorescent confocal microscopy of aquaporin 0 in zebrafish wild type and lenses at 60 hpf shows that the expression of this lens fiber cell marker confined to the lens (a, b). In contrast, aquaporin 0 expression is detected both in the malformed lens and in material extruding out of the eye anteriorly in the mutants (c, d). Red: aquaporin 0; blue: Draq5. f: lens fiber cells; ce: corneal epithelium. Scale bar = 35 *μ*m.

The lens expresses the laminin receptors *α*6*β*1, *α*6*β*4, and *α*3*β*1 integrin and mice lacking either both the *itga3* and *itga6* or *itgb1* genes from the lens develop profound lens abnormalities including loss of the lens epithelium and fiber cell defects [5, 65–67]. Further, lens cells grown in vitro are commonly cultured on laminin to allow for their survival in serum free culture [68], while laminin/*α*6*β*1 integrin interactions are necessary for fiber cell differentiation in vitro [69]. Since defects in lens fiber cell differentiation have been proposed to cause the lens defects in *lama1* ^*a69/a69*^ mutants, we evaluated these lenses for the expression of lens fiber cell markers. Aquaporin 0 is the most abundant membrane protein found in vertebrate lens fiber cells [70] that serves as both a water channel and cell adhesion molecule necessary for fiber cell physiology [71]. In the zebrafish, aquaporin 0 is encoded by two genes (*aqp0a* and *aqp0b*), and both initiate mRNA expression in the lens at 22 hpf, and this expression is maintained at high levels throughout development [72]. Consistent with this, an aquaporin 0 antibody expected to react similarly with both zebrafish isoforms robustly labels the lens fiber cell membranes but not the lens epithelium of 60 hpf wildtype zebrafish lens fiber cells (Figures 3(a) and 3(b)). Importantly,* lama1* ^*a69/a69*^ mutant lenses also stain robustly for aquaporin 0, although the distribution is more disorganized reflecting the morphological defects seen in these lenses [26] (Figures 3(c) and 3(d)). Notably though, clusters of aquaporin 0 positive cells were routinely detected adhered to the outer surface of the developing cornea suggesting that while fiber cell differentiation per se is not affected in this mutant, the lens is rupturing through the cornea (Figures 3(c) and 3(d) arrowheads). Similarly, staining lenses with the monoclonal antibody, ZL1, which recognizes a marker of zebrafish fiber cell differentiation which is first expressed in the lens between 20 and 23 hpf. [73], showed that the lens fibers of *lama1* ^*a69/a69*^ mutants appropriately entered the lens fiber cell differentiation pathway although their structural organization is abnormal.

Currently, the role of laminin in regulating the differentiation of lens fiber cells is unclear. The observation that lens fiber cell marker expression in *lama1* ^*a69/a69*^ mutants is preserved despite the morphological abnormalities seen in these lenses is consistent with a prior report showing that Zl-1 expression is retained in *lamc1* mutant lenses [18]. However, experiments utilizing chick lens cultures and microdissected embryonic lenses have found that lens cells undergo optimal differentiation when plated on laminin that the expression and cytoskeletal linkage of *α*6-integrin, a component of *α*6*β*1 and *α*6*β*4 integrin, the most abundant laminin receptors in the lens, changes during fiber cell differentiation and knockdown of *α*6 integrin expression in cultured LECs blocks their differentiation into fibers [74]. In contrast, *β*1-integrin is necessary for the maintenance of the mouse lens epithelium with its loss corresponding to the upregulation of some lens fiber cell markers and the EMT marker *α*-smooth muscle actin followed by epithelial cell apoptosis. While it has been proposed that *β*1-integrins are also important for lens fiber cell survival [66], conditional deletion of *β*1-integrin from lens fibers leads to defects in lens fiber cell structure, but not lens fiber cell survival or differentiation per se [67]. These data in aggregate lead to the proposition that laminin interactions with integrins expressed by lens cells are important for the proper morphological organization of lens fibers, with the caveat that both *α*6 and *β*1 integrin are also localized to the lateral membranes of lens fibers away from the laminin of the lens capsule and may be playing roles independent of their function as laminin receptors [67, 74].

### 3.4. Laminin Mutant Zebrafish Have Defects in Corneal Integrity

The loss of the lens capsule and lens fragmentation seen in *lama1* ^*a69/a69*^ (Figures 1 and 2) as well as *lamb1* and *lamc1* mutants [18] implies that laminin 111 is important to form the lens capsule and is consistent with our prior understanding of the role of the lens capsule in the maintenance of lens structural integrity [1, 19]. However, we also routinely observed that a portion of the lens fiber mass extruded to the exterior of the cornea by 60 hpf, indicating that the structural integrity of the cornea was also compromised.

**Figure 4:**
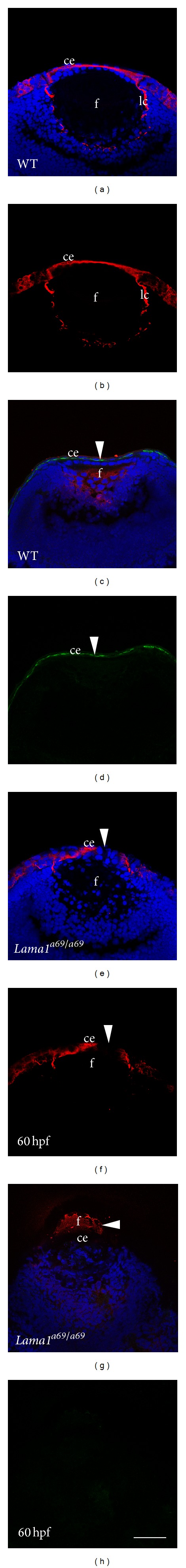
Laminin and BIGH3 expression downregulates in the developing cornea of *lama1* ^*a69/a69*^ mutant zebrafish. immunofluorescent confocal microscopy showing normal expression and distribution of laminin at 60 hpf in wild type embryos (a, b). Zebrafish *lama1* ^*a69/a69*^ mutants show downregulation of laminin in lens capsule (e) and discontinuous laminin staining in the developing cornea ((f) arrowheads). BIGH3 costaining with lens fiber cell specific marker ZL1 shows normal distribution at 60 hpf, in wild type embryos (c, d). Zebrafish *lama1* ^*a69/a69*^ mutant embryos show downregulation of corneal BIGH3 (h) and ZL1 positive cells were detected anterior to the anatomical boundary of the eye ((g) arrowheads). Laminin: red; (a, b, e, f), ZL1: red (c, d, g, h); BIGH3: green (c, d, g, h); Draq5: blue. f: lens fiber cells; ce: corneal epithelium; lc: lens capsule. Scale bar = 35 *μ*m.

Immunolocalization using a pan-laminin antibody revealed that at 60 hpf, laminin was found both in the lens capsule as well as the basement membrane underlying the developing corneal epithelium (Figures 4(a) and 4(b)). This staining was absent from the region surrounding the *lama1* ^*a69/a69*^ lens as expected, while some laminin immunoreactivity was still detected underlying the corneal epithelium, although it was discontinuous (Figures 4(c) and 4(d) arrowheads) suggesting that the corneal BM structure is compromised. The laminin composition of the zebrafish corneal BM has not been reported; however, in humans, lam*α*3 and lam*α*5 are found to be the predominant laminin *α* chains in the BM underlying the adult corneal epithelium, while lam*α*1 was not detected [22]. Thus, lam*α*1 may be necessary for the initial organization of the corneal BM but later in development, it is replaced by other laminin *α* proteins. This would be consistent with the observation that laminin 111 is deposited early in the development of most epithelia, although in most cases it is replaced by other laminins later in development [55].

Transforming growth factor, beta-induced (TGF*β*i, BIGH3) is an extracellular matrix protein first named for the induction of its expression by transforming growth factor *β* [75, 76]. Mutations in this gene result in a variety of human corneal dystrophies and its expression has been detected in the developing cornea of mice, rabbits, and zebrafish [77, 78]. In the cornea, it is found beneath the corneal epithelium associated with the BM where it serves as an adhesion matrix for the epithelial cells [79]. TGF*β*i interacts with several ECM components such as collagen, fibronectin, and laminin and this interaction is important for the maintaining integrity of the corneal epithelium by inhibiting cell migration and promoting cell-cell and cell-ECM adhesion [80]. Since *lama1* ^*a69/a69*^ mutants have defects in the BM underlying the presumptive corneal epithelium and exhibited an extrusion of lens fiber cells anteriorly, we sought to determine whether TGF*β*i was appropriately found in the developing cornea. TGF*β*i was detected in a discrete line below the corneal epithelium (green) in wildtype eyes (Figures 4(e) and 4(f) arrowheads) while the zebrafish lens marker Zl-1 was confined to the lens fiber cells (Figure 4(f)) at 60 hpf. However, TGF*β*i was not detected in *lama1* ^*a69/a69*^ eyes (Figure 4(h)), and cells staining with Zl-1 were found outside of the anatomical boundaries of the eye (Figure 4(g) arrowheads) compared to the wild type zebrafish embryos (Figures 4(e) and 4(f)). These data in aggregate show that the structural integrity of the corneal epithelium is disrupted in *lama1* ^*a69/a69*^ mutants, suggesting that laminin 111 is playing both structural and signaling functions in the developing zebrafish eye.

## 4. Conclusion

Our data demonstrate that lam*α*1 is essential for the formation of the lens capsule including the deposition of collagen IV into the capsule and thus lens morphology/structure. Further, lam*α*1 is essential for the organization of the corneal epithelium including deposition of TGF*β*i underneath the corneal epithelium. These data suggest that the *lama1* ^*a69/a69*^ mutant phenotype is due to a combination of both a structural and signaling function of the lens capsule and early corneal epithelial BM during early eye development.
